# ‘Saying goodbye’. . . A systematic integrative review of palliative caregiving in intergenerational living contexts

**DOI:** 10.1177/02692163251394880

**Published:** 2025-12-01

**Authors:** Madeleine L Juhrmann, Priyanka Vandersman, Raechel A Damarell, Ahmed Khamis Sharaf, Aljon San Martin, Andrew Donkor, Yakubu Salifu

**Affiliations:** 1 Research Centre for Palliative Care, Death and Dying (RePaDD), College of Nursing and Health Sciences, Flinders University, Bedford Park, SA, Australia; 2 Improving Palliative, Aged and Chronic Care through Clinical Research and Translation (IMPACCT), University of Technology Sydney, Ultimo, NSW, Australia; 3 The Palliative Centre, HammondCare, Greenwich, NSW, Australia; 4 Community Health Nursing Department, Faculty of Nursing, Alexandria University, Egypt; 5 Susan Wakil School of Nursing, Sydney Nursing School, Faculty of Medicine and Health, The University of Sydney, Camperdown, NSW, Australia; 6 Department of Medical Imaging, Faculty of Allied Health Sciences, Kwame Nkrumah University of Science and Technology, Kumasi, Ghana; 7 International Observatory on End-of-Life Care (IOELC), Division of Health Research, Lancaster University, UK

**Keywords:** intergenerational relations, extended family, caregivers, palliative care, terminal care

## Abstract

**Background:**

Intergenerational co-residence has historically been prevalent. Recent financial pressures, increasing caregiving responsibilities and ageing populations have led to a resurgence of this arrangement, particularly in end-of-life care. However, understanding of its influence on care quality across cultures remains limited.

**Aim:**

To explore how intergenerational co-residence affects emotional, practical, and cultural dimensions of palliative and end-of-life care across different settings, and to identify key themes shaping caregiving roles, decision-making, and support needs in these households.

**Design:**

Systematic integrative review and thematic synthesis based on Braun and Clarke’s approach and using the Convoy Model as a theoretical framework. PROSPERO ID: CRD42023446688.

**Data sources:**

Six major databases were searched from inception to 22 May 2023 and updated to 8 June 2025. Eligible papers reported empirical studies on end-of-life care in intergenerational co-residence and were appraised using the Mixed Methods Appraisal Tool.

**Results:**

Three themes were constructed from seven studies from China, South Africa, Spain, Uganda, Turkey and the United States. These were: responding to an end-of-life diagnosis, with limited death literacy delaying care; identifying systems of support, where caregiving burdens often fell on women; and concluding the journey and saying goodbye.

**Conclusions:**

Intergenerational co-residence can foster support at the end of life, yet it may also reinforce gendered caregiving roles that disproportionately burden women. Effective integration of formal support services with family caregiving remains important in alleviating pressures and promoting equitable care models, highlighting the need for culturally sensitive interventions that address the diverse needs of families, while encouraging collaborative caregiving approaches.

**What is already known about the topic?**

- Intergenerational co-residence has long served as a foundational family structure, particularly in Eastern cultures, providing essential emotional and practical support in caregiving contexts.
- Current literature highlights a resurgence of intergenerational living arrangements in response to financial pressures, evolving family dynamics and increasing caregiving responsibilities among ageing populations.
- Despite these trends, there is still limited research on the implications of intergenerational co-residence on the quality and delivery of palliative and end-of-life care, particularly in Western contexts that emphasise individualism.

**What this paper adds?**

- The review indicates intergenerational co-residence may enhance emotional support and continuity of care and could play an important role in facilitating a more holistic approach to end-of-life care by leveraging familial relationships.
- The findings highlight the dual nature of these arrangements; while they provide essential support, they also perpetuate gendered caregiving roles, often placing disproportionate burdens on women and raising concerns about caregiver burnout and inequity.
- The review suggests the successful integration of formal support services with family caregiving is vital in addressing caregiver strain and fostering sustainable, compassionate palliative care models that honour family dynamics.

**Implications for practice, theory or policy**

- Insights from this review suggest a pressing need for culturally tailored interventions sensitive to the diverse needs and challenges of intergenerational households, ensuring that care is both effective and respectful of cultural norms.
- Policymakers should consider developing frameworks empowering family caregivers—especially women—through comprehensive training, accessible resources and financial support, recognising their vital role in the end-of-life caregiving continuum.
- Future research could delve deeper into the complexities of intergenerational dynamics within various cultural settings, aiming to identify best practices that can enhance the effectiveness and accessibility of palliative care services.

## Background

Intergenerational co-residence—defined as three or more generations of a family living under one roof—has historically played a vital role in many societies. In recent decades, changing economic conditions, evolving family structures and demographic shifts such as population ageing have led to renewed interest in this living arrangement. Across different cultural contexts, intergenerational living can provide critical social, emotional and practical support, particularly in times of illness or caregiving need.^1–3^ This arrangement holds unique significance in the context of palliative and end-of-life care.

As health systems globally promote home-based models of care, families are increasingly expected to absorb the burden of support traditionally delivered by professionals. Within intergenerational households, these responsibilities are often negotiated through complex cultural norms, gender roles and expectations of filial duty. However, the dynamics of care provision in intergenerational settings remain under-researched, especially regarding how caregiving responsibilities are distributed, how decisions are made and how emotional labour is shared.

In the last decade, younger generations have increasingly turned to intergenerational living arrangements due to the convergence of ageing populations, increasing caregiving responsibilities, and rising costs of living.^4^ A 2021 survey showed that 26% of Americans now live in multigenerational households, up from 7% in 2011.^5^ As the elderly population grows, the demand for home-based care has increased, often resulting in younger family members providing support within the same household.^4^ Simultaneously, the financial pressures of housing and everyday expenses have made multigenerational living a pragmatic solution, allowing families to pool resources and share caregiving duties more effectively.^4,5^

While certain collectivist values are common in some East Asian cultures, there is significant variation across countries and communities. Studies suggest that in Confucian-influenced societies, such as Japan and Korea, family-centred decision-making often guides healthcare and end-of-life choices.^6,7^ Conversely, in many Anglo-American contexts, including the UK and the US, there is a stronger emphasis on individual autonomy and advance care planning.^8,9^ These cultural differences profoundly shape who provides care, how care is given and what constitutes a ‘good death’.^10–14^ Yet, these general patterns do not always reflect the lived realities of families, especially in settings where migration, economic hardship, or systemic inequality disrupt traditional caregiving structures.^15,16^For example, Indigenous communities within Western societies may blend collectivist values with community-based approaches to care, challenging East-West dichotomies.^17,18^

Intergenerational co-residence has positive implications for families, including strengthening family bonds, improving personal mental health and facilitating the sharing of caring responsibilities among family members.^2,19^ These living arrangements are especially supportive for families when a member is diagnosed with a life-limiting illness and requires palliative and end-of-life care.^20,21^ This proximity can foster a supportive environment that meets the emotional, physical, and practical needs of the care recipient.^22^ Some studies also suggest that palliative and end-of-life care in the community and home deaths are more preferred and attainable with the presence of family caregivers in the same household.^23–25^ However, intergenerational co-residence can also create family tension due to compromising personal routines, issues of privacy, and caregiver distress.^2,19^ Hence, the literature demonstrates family dynamics play a critical role in the provision of end-of-life care at home.^24^

Despite growing interest in informal caregiving and cultural competency in palliative care, the specific intersection between intergenerational living arrangements and palliative care remains poorly understood. Most studies to date have focused on individual caregivers or nuclear family settings, neglecting the relational complexity of intergenerational households. This gap limits our understanding of caregiving dynamics, role negotiation and the emotional and structural supports available in these households. To our knowledge, this is the first systematic integrative review to explore intergenerational co-residence as a structural and cultural factor shaping palliative and end-of-life care across diverse settings. In doing so, this study addresses a critical gap in the palliative care literature and offers new insights into how family living arrangements affect caregiving processes, outcomes, and equity.^26,27^

This review addresses that gap by systematically examining global literature on intergenerational co-residence and its role in shaping end-of-life care. In doing so, it asks:

1. How do caregiving roles and responsibilities emerge and evolve in intergenerational households?
2. What cultural, relational, and structural factors influence care delivery and decision-making at the end of life in such settings?

Consequently, this review aimed to:

(1) Explore the empirical evidence on how intergenerational co-residence impacts the provision and experience of palliative and end-of-life care;
(2) Examine the roles, responsibilities and relational dynamics among family members in such contexts; and
(3) Identify cultural, gendered and systemic factors that influence the quality and equity of care delivery within intergenerational households.

## Methods

This study employed a systematic integrative review design, allowing the inclusion and synthesis of empirical studies with qualitative, quantitative and mixed methods approaches. This design was chosen to comprehensively capture and interpret the multidimensional experiences of intergenerational caregiving at the end of life.^28^ The methods are described according to the Preferred Reporting Items for Systematic reviews and Meta-Analyses (PRISMA) statement.^29^ The protocol was registered with the PROSPERO (CRD42023446688). The eligibility criteria are reported in Table 1.

**Table 1.** Inclusion and exclusion criteria.

Category	Inclusion criteria	Exclusion criteria
Population	Stakeholders (patients, families, healthcare professionals or policymakers) experiencing or providing intergenerational care (determined as at least three generations living together in the same household).	Non-intergenerational household care.
Interest	Experiences, perceptions or attitudes on palliative and end-of-life care in intergenerational living.	Studies not exploring the impact of intergenerational care.
Study types	All types of empirical studies of any design in the following languages: English, German, Nepalese, Hindi, Arabic, and Tagalog at any publication date.	Editorials, commentaries and abstracts were excluded. Languages other than English and those listed in the inclusion set.
Context	Intergenerational co-residence in any setting.	Studies that do not indicate intergenerational co-residence circumstances.

### Search strategy

Published sources were explored using a search strategy involving database searches, manual checking of the reference lists of included studies and grey literature searching using Google Advanced.

## Database searches

Databases were searched from inception to 22 May 2023, and updated to 8 June 2025, using search strings comprising a wide range of synonyms describing two concepts: (1) intergenerational living and (2) palliative/end-of-life care. The search was first developed in the Ovid Medline database and then translated for the following additional five databases: Embase (Ovid), CINAHL (EBSCOhost), APA PsycINFO (Ovid), Applied Social Science Index & Abstracts: ASSIA (ProQuest), and the Web of Science Core Collection. Language limits were applied in accordance with the eligibility criteria and bilingual reviewers involved. No date limits were applied. Full search strategies are provided as Supplemental Table 4.

Citations identified by the search strategies were exported into an EndNote Library (version 20) where duplicates were identified and removed.

## Grey literature search

Iterative Google (Advanced mode) searches were conducted by successively combining each of the terms ‘end of life’, ‘palliative’ and ‘life limiting’ with the search strings describing ‘Intergenerational living’. For each of the three search iterations, the first 100 retrievals were checked for relevance.

### Study selection

Deduplicated citations were uploaded into Covidence for screening (RD, PV, ASM).^30^ Eligibility criteria were first piloted by the three researchers who independently reviewed titles and abstracts of the first 100 citations retrieved, discussing and resolving any discrepancies in interpretation or areas of ambiguity. This same team then went on to dual screen all retrieved citations, before working as a group to resolve conflicts. An additional researcher, MJ, was brought in once where a particular language skill was required. The same three researchers then independently screened full-text articles for inclusion. Again, in case of disagreement, consensus on eligibility was reached through discussion.

### Data extraction, analysis and syntheses

The data extraction and quality appraisal processes were carefully structured to ensure rigour and reliability throughout the systematic review. The JBI data extraction tool was utilised to systematically capture relevant information from each study, including study characteristics, population, interventions, outcomes and key findings.^31^ Two independent reviewers, MJ and AD, conducted the extraction separately to avoid bias. After completing their independent reviews, the data were compared, and any discrepancies were discussed and resolved, working towards 100% inter-rater reliability.

The quality appraisal followed a similar process, employing the MMAT 2018 tool to evaluate the methodological quality of the studies included in the review.^32^ This tool was selected for its suitability in reviews that include qualitative, quantitative, and mixed methods studies. The MMAT allows for a consistent and comparable assessment across diverse study designs, ensuring that methodological rigour is evaluated within a single integrated framework while still addressing design-specific considerations. Two authors, YS and AKS, conducted the quality appraisals independently and later compared their results to reach consensus, ensuring full agreement and further reinforcing the validity of the review.

Consistent with an integrative review methodology, diverse empirical studies were analysed through thematic synthesis, drawing on Braun and Clarke’s six-step reflexive thematic analysis process.^33^ This flexible yet rigorous method enabled us to explore and interpret patterns across multiple methodologies and contexts. Data analysis was conducted by two authors, MJ and AD, and followed Braun and Clarke’s six-step reflexive thematic analysis process,^33^ beginning with familiarisation with the data, followed by systematic coding. From these codes, themes were constructed, reviewed, and refined to ensure consistency with the data set, and then named to represent key patterns. Investigator triangulation was employed, with both analysts independently coding data before comparing and discussing interpretations to reach consensus. Reflexivity was maintained through regular team discussions, and analytic decisions were documented to minimise interpretive bias.

### Theoretical framework

The Convoy Model of Social Relations^34^ informed this iterative approach, whereby it conceptualises social relationships as dynamic networks providing varying levels of support over the life course. This model informed data analysis by sensitising the team to structural, functional, and contextual dimensions of caregiving networks. Codes and themes were interpreted through this lens, enabling exploration of how caregiving roles, resources and relational patterns in intergenerational households evolve in the context of end-of-life care. A coding tree (Supplemental Table 5) was developed to visually represent how initial codes were refined into subthemes and final themes.

## Results

**Figure 1.**
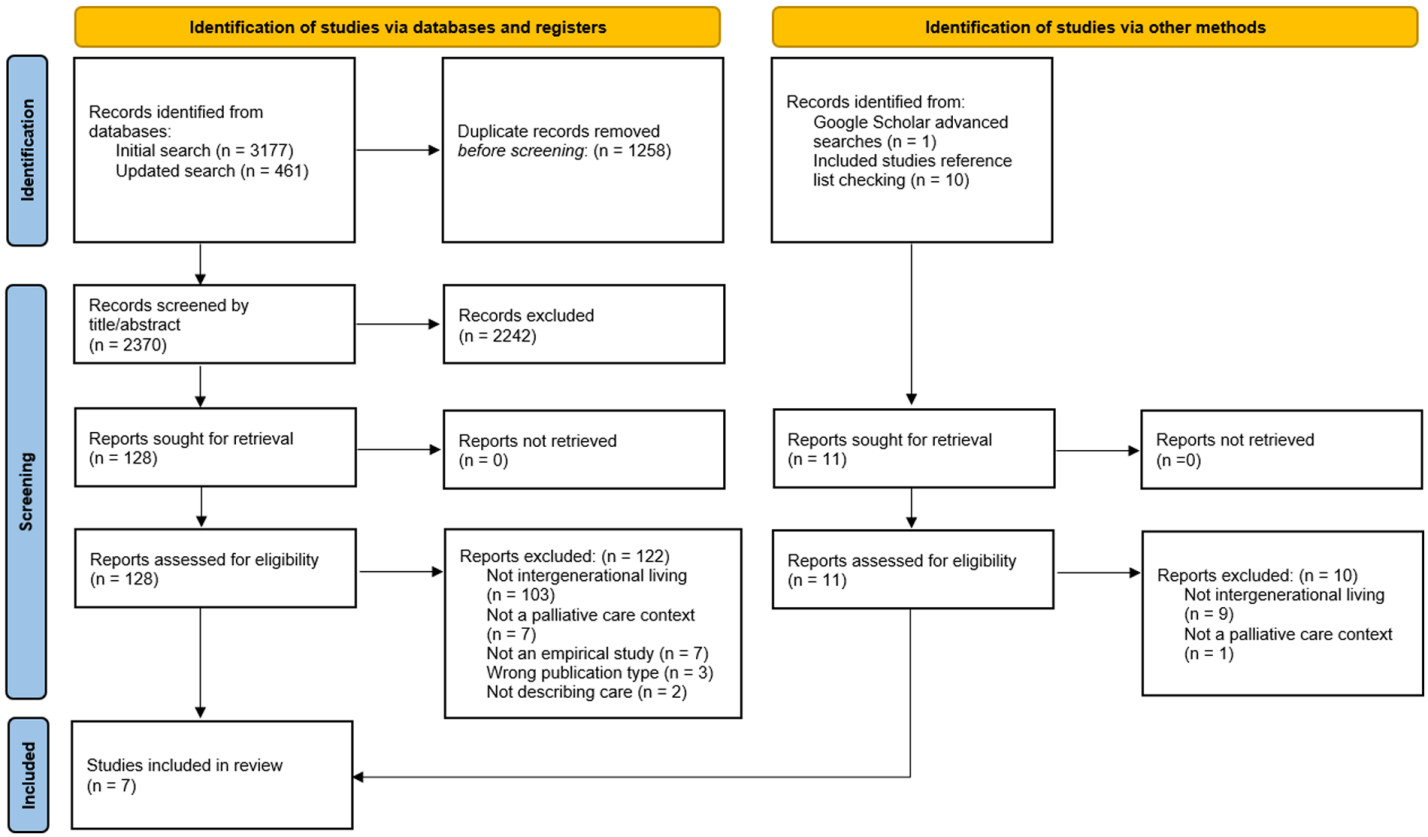
PRISMA flow chart.

Database searches retrieved a total of 3177 records, of which 2026 remained for screening once duplicates were removed. Based on a full text reading, six studies met eligibility criteria. No relevant grey literature was identified. An additional study was included based on reference list checking of the included papers. This selection process is presented graphically in the form of a PRISMA flow chart (Figure 1), the study characteristics are outlined in Table 2, and the quality appraisal illustrated in Table 3.^29^

**Table 2.** Data extraction.

Author_Year (country)	Journal	Type of study (methods)	Participant details	Phenomena of interest	Setting and other context related information	Outcomes or findings of significance to the review objectives	Author’s conclusions
Bryer_1979 (USA)	American Psychologist	Qualitative ethnographic study (Interviews and observations)	(*n* = 24), Amish families living in Pennsylvania	The study focused on five key areas: family structure, group structure, funeral customs and mourning rituals, personal experiences with death, and personal feelings about death. It aimed to highlight how these factors influence coping with death within the Amish	Amish families living in Pennsylvania	Key factors that help cope with death in the Amish society were identified: • Constant presence of family members throughout the illness and at the time of death. • Open discussions about dying and its effects on the family. • Maintaining a normal lifestyle during the illness. • Promoting the independence of the dying person as much as possible. • Allowing the dying individual to plan and organise their own death. • Providing continued bereavement support for at least a year after the funeral, with ongoing care for those who do not remarry. These practices reflect the importance of family cohesion, open communication, and long-term emotional support in Amish culture when managing end-of-life situations.	• Death is viewed as a natural part of life in Amish society. • Strong family and community systems help individuals cope with loss. • Family members are present during illness and death. • Open communication about dying is encouraged. • Maintaining the independence of the dying person is important. • Individuals are encouraged to plan and organise their own death. • Separation from family during the dying process can cause distress. • Bereaved individuals receive support for at least a year after the funeral. The Amish approach, rooted in religious and communal values, allows for a calm acceptance of death, ensuring that both the dying person and their family are supported throughout this experience.
Cong and Silverstein_2015 (China)	Canadian Journal of Aging	Quantitative study (Surveys)	(*n* = 1715), Adults aged 60 years or older living in rural townships	To explore whether the propensity of older parents to live with their sons at the time of death has changed over historical time and whether past intergenerational exchanges promote co-residence.	Rural area of China’s Anhui province	Bequest Decisions: • Parents who left all assets to children or made no bequest were 5–6 times more likely to live with a son (OR = 5.24 and 5.93). Instrumental Support: • Receiving support from sons increased co-residence likelihood nearly twofold (OR = 1.86). • Support from daughters had no effect on co-residence with sons. • Providing support to sons increased co-residence likelihood almost threefold (OR = 2.94). • Providing support to daughters had no effect. Emotional Closeness: • Closeness with sons increased likelihood of co-residence (OR = 1.29). • Closeness with daughters decreased it (OR = 0.81). Grandchild Care: • Caring for a daughter’s children significantly increased co-residence with a son (OR = 4.29). • Caring for a son’s children had no effect. Number of Sons: • Having more sons decreased the chance of living with any one son (OR = 0.71). Other Variables: • Not statistically significant in predicting co-residence.	• Adult sons continue to move in with parents at the end of life. • This practice is weakening in rural China due to modernisation. • Despite a rise in co-residence before death, overall trends show a decline. • Neo-local arrangements (shared care without co-residence) are becoming more common. • Parents’ earlier investments in sons reinforce expectations of end-of-life co-residence. • Sons who provided earlier support may feel a stronger sense of filial duty. • Parents who received support from sons may expect more intensive end-of-life care. • Strong emotional bonds with daughters may reduce reliance on sons. • Emotional closeness with daughters can weaken traditional co-residence patterns with sons.
de Graaff and Francke_2003 (Netherlands)	International Journal of Nursing Studies	Qualitative study (Semi-structured interviews)	(*n* = 19), Relatives of elderly terminally ill Turks and Morocccans living in the Netherlands	To explore the experiences of relatives of elderly terminally ill Turks and Moroccans regarding Dutch professional home care and the barriers to the use this care.	Relatives of elderly terminally ill Turks and Morocccans living in the Netherlands	There is no consistent pattern in the use of home care among Turkish and Moroccan families in the Netherlands. Families who used home care reported satisfaction, especially with support and equipment. Factors for home care use: • Nature of illness • Family structure • Family decision-making processes • Community pressures • Access to information about services • Formal referrals from professionals • Strong preference for family-based (informal) care due to cultural expectations. Systemic Implications: • Healthcare systems need to address cultural and informational barriers. • Support is needed to help families balance formal and informal caregiving.	• Turkish and Moroccan families using Dutch home care are generally satisfied. • Equipment and nursing support are especially appreciated. • Nurses are viewed as more culturally sensitive than general practitioners. • Cultural norms prioritise family-based care, especially among men. • Preference for dying in the home country limits use of Dutch home care. • Strong community expectations discourage external care in some areas. • In areas with fewer Turkish and Moroccan families, social pressure is weaker. • These areas show greater acceptance of professional home care. • Women are more open to using professional care than men. • Despite large family networks, caregiving often falls on one female member. • This caregiving role is demanding and stressful. The study highlights the need for culturally tailored care and better communication to support these families.
Karabayir and Ar-Karci_2022 (Turkey)	Current Psychology	Qualitative study (Semi-structured interviews)	(*n* = 7), College students	To examine the impact of grandparental loss on Turkish college students from a developmental and cultural perspective.	Turkish universities and communities	Five significant themes related to the grief process experienced by individuals following grandparental loss. • The loss of a grandparent was often perceived as the loss of a second parent, intensifying the grief. • Experiencing death for the first time was transformative, reshaping perspectives on life and death. • Individuals expressed ambivalence towards death-related rituals, struggling with their meaning and relevance. • Grandparental loss triggered a dual grief process, as individuals mourned both the loss of the grandparent and their childhood memories. • The university environment served as both a barrier and a refuge, offering distraction from grief while also limiting the support network. These findings highlight the complex, multifaceted nature of grieving a grandparent and suggest the need for targeted support in such contexts.	• Grandparent loss multifaceted and emotionally challenging experience and often felt as a second parental loss. • This intensifies the grieving process for many individuals. • In Turkey, grandparents significantly influence physical and psychosocial development. • Loss of unique relationships and family unity are key culture-related grief factors. • Being away from family and identity exploration can complicate grief during university years. • Personalised grief counselling should be sensitive to discomfort with public mourning. • University counselling services should offer both individual and group support. • Time- and cost-effective intervention programmes are recommended for bereaved students.
Martin-Martin et al_2022 (Spain)	Journey of Family Nursing	Qualitative study (Semi-structured interviews)	(*n* = 9 families and 21 individuals), caring for ill family members	To understand families’ unitary experiences of providing home care to terminally ill family member.	Families who cared for a terminally ill family member at home and were cared for by the Primary Care System of the city of Pamplona, Spain	Six key themes emerged from the experiences of families caring for an ill family member. • Families adapt to a new life focused on caregiving, becoming the centre of their daily routines. • Families emphasise the importance of collective support from all family members living in the home to meet these care demands. • Providing care is seen as a moral duty, bringing emotional satisfaction. • Families experience ambivalence about their loved one’s impending death, simultaneously acknowledging that death may bring relief while instinctively wanting to preserve life. • Caregiving disrupts family well-being, affecting physical, psychological, social, and interpersonal relationships. • Families feel unsupported by socio-health systems, lacking guidance on resources and financial aid, with professionals focusing mainly on the patient.	• To help cope with emotional strain, families strive to establish a new sense of normality centred on caregiving. • Families frequently experience conflicting emotions about the impending death of their loved one, and open conversations about these feelings are essential. • Caregiving involves physical and emotional strain. • Families must adapt to new routines and responsibilities. • Collaborative caregiving helps manage the demands more effectively. • More studies are needed to explore psychological support strategies for families during end-of-life care.
Munthree and Maharaj_2010 (South Africa)	HIV/AIDS and Older Persons	Mixed-methods study (Surveys and focus groups)	(*n* = 974 surveys) and 8 focus groups (6–8 people), Households effected by HIV/AIDS	To explore the multiple impacts of AIDS on older people in the context of a high prevalence of HIV/AIDS. This article seeks to provide insight into the experiences of older persons, with a particular focus on their roles and activities in households affected by HIV/AIDS, and to gain understanding about how older people are responding to the challenge of HIV/AIDS in their daily lives.	Conducted in urban and rural sites of KwaZulu-Natal—the province with one of the highest rates of HIV infection in South Africa. AIDS is the leading cause of adult mortality in KwaZulu-Natal, accounting for 73% of female and 61% of male deaths at ages 15–44 year.	Older men and women providing care for individuals with HIV/AIDS often feel at risk of contracting the virus. Nearly 17% of respondents indicated they had cared for someone with HIV/AIDS, with slightly higher rates in rural areas where healthcare resources are less accessible. Physical, emotional, and financial strain is intensified by gender roles and limited resources, with older women disproportionately responsible for caregiving. The findings highlight the need for targeted interventions to address the risks and challenges faced by caregivers, particularly in rural and under-resourced areas.	• Elderly women in South Africa are culturally expected to be caregivers in intergenerational households. • This role places a significant physical and emotional burden on them. • Lack of formal support systems and financial assistance worsens the strain. • Older caregivers struggle to balance caregiving duties with their own well-being. • The absence of structured support leaves families vulnerable and overwhelmed. • The healthcare system needs to take a more active role in supporting caregivers. • Targeted interventions like financial aid and psychological services are recommended. • Policies and programmes should be developed to support caregivers in intergenerational households.
Ssengonzi_2007 (Uganda)	Journal of Cross-Cultural Gerontology	Qualitative study (Surveys and focus groups)	(*n* = 20 interviews and 20 focus groups), Parents, relatives and/or caregivers of persons infected by HIV/AIDS	To describe the challenges faced by the elderly as caregivers of people infected/affected by HIV/AIDS and the implications of such challenges on the well-being and on the ageing process of these older persons.	10 rural and urban communities within two Ugandan districts of Luwero and Kamuli	• The elderly provide essential care to patients with AIDS at the terminal stage, when constant care is most needed. • This responsibility often extends to caring for children affected by HIV/AIDS, beginning before the children are orphaned. • This demanding caregiving role negatively impacts the elderly across economic, emotional, physical, and nutritional dimensions, affecting their overall health and well-being. • Elderly women, who bear the bulk of caregiving duties, report higher rates of physical ailments compared to men, reflecting their disproportionate caregiving burden. • Many elderly caregivers also experience significant anxiety about their future health, largely due to the pressures of the HIV/AIDS epidemic. • These challenges exacerbate the ageing process, especially given the poor resources and weak healthcare infrastructure in their environment.	• Elderly caregivers, especially women, bear a disproportionate burden in caring for AIDS patients and children affected by HIV/AIDS. • This role significantly impacts their physical, emotional, and economic well-being. • Caregiving responsibilities accelerate the ageing process in elderly caregivers. • Weak health infrastructure and limited community resources worsen these effects. • There is an urgent need for policy interventions to support elderly caregivers. • Strengthening support systems is essential, particularly in the context of the HIV/AIDS epidemic.

**Table 3.** Quality appraisal.

Study design: qualitative									
Author_Year (Country)	S1 – Are there clear research questions?	S2 – Do the collected data allow to address the research question?	1.1 – Is the qualitative approach appropriate to answer the research question?	1.2 – Are the qualitative data collection methods adequate to address the research question?	1.3 – Are the findings adequately derived from the data?	1.4 – Is the interpretation of results sufficiently substantiated by data?	1.5 – Is there coherence between qualitative data sources, collection, analysis and interpretation?	Total score	Comments
Bryer_1979 (USA)	Yes	Yes	Can’t tell (No clear discussion about the used approach as it was implied from the interview methods used and the way of reporting	Can’t tell (No adequate discussion about the layout of the interview/how it was conducted and whether it was recorded)	Can’t tell (No discussion about how data was analysed)	Yes (But there is minimal use of participant’s quotes)	Yes (However, no clear discussion about the data analysis methods)	2	In this article, there is no clear discussion about the qualitative approach used as it was implied from the methods used (interview) and the way of reporting. No adequate discussion was provided about the layout of the interview, how it was conducted and whether recorded or not. There is a limited use of Participant quotes. There is no clear discussion about the data analysis methods.
De Graff and Francke_2003 (Netherlands)	Yes	Yes	Yes	Yes	Yes	Yes	Yes	5	This is a good article that outlined the justification of methods used, and details about the data collection and analysis process, however, there is little use of participant’s quotes in the findings section.
Karabayir and Ar-Karci_2022 (Turkey)	Yes	Yes	Yes (Justification was provided in the article of using IPA)	Yes	Yes (The data analysis process was clear and an explanation regarding how themes were developed was provided)	Yes (Use of necessary participant’s narratives)	Yes	5	This is a good article that outlines the used methodological approach with proper justification, a strong explanation of methods and the use of strategies to enhance the trustworthiness of the findings. There is a good discussion of findings and links with the current literature, with elaboration on practical and research implications.
Martin-Martin et al_2022 (Spain)	Yes	Yes	Yes (Justification was provided)	Yes	Yes	Yes	Yes	5	This is a good article that outlined the justification of methods used, details about the data collection and analysis process and data were represented by adequate participant quotations
Ssengonzi, 2007, Uganda	Yes	Yes	Yes (However, no clear justification was provided about using the qualitative methods)	Yes	Yes (the analysis process was discussed clearly)	Yes (However, some late themes have no quotes illustration such as nutritional and physical impact)	Yes	5	This is a good article, however, there is no clear justification for the use of qualitative methods. There is a limited use of participant quotes in the late themes such as nutritional and physical impact.
Study design: quantitative descriptive									
Author_Year (Country)	S1 – Are there clear research questions?	S2 – Do the collected data allow to address the research question?	4.1 – Is the sampling strategy relevant to address the research question?	4.2 – Is the sample representative of the target population?	4.3 – Are the measurements appropriate?	4.4 – Is the risk of non-response bias low?	4.5 – Is the statistical analysis appropriate to answer the research question?		
Cong and Silverstein_2015 (China)	Yes	Yes	Yes (Use of probability technique)	Yes (High response rate 95.3%)	Yes (Variables were clearly defined)	Yes (High response rate 95%)	Yes (Use of logistic regression to identify predictors of older parents’ residence with sons before death in rural China)	5	This is a good article; probability techniques were used for sampling methods with a high response rate of the participants. Variables are clear and logistic regression was useful to define the predictors of residence of older parents with sons before death in rural China.
Study design: mixed methods									
Author_Year (Country)	S1 – Are there clear research questions?	S2 – Do the collected data allow to address the research question?	5.1 – Is there an adequate rationale for using a mixed methods design to address the research question?	5.2 – Are the different components of the study effectively integrated to answer the research question?	5.3 – Are the outputs of the integration of qualitative and quantitative components adequately interpreted?	5.4 – Are divergences and inconsistencies between quantitative and qualitative results adequately addressed?	5.5 – Do the different components of the study adhere to the quality criteria of each tradition of the methods involved?		
Munthree and Maharaj_2010 (South Africa)	Yes	Yes	Yes	Yes	Yes	Yes (No divergences)	Yes	5	This is a good article in the Quantitative part) (Survey): data was obtained from a household survey through a random sampling technique used with adequate sample size and response rate. The use of descriptive statistics was useful to define the sample characteristics. The use of logistic regression was useful in of logistic regression for defining factors of High, Medium HIV Infection) The qualitative part was used to complement the quantitative part. The use of focus groups was justified and analysis process was discussed clearly. The integration of qualitative and quantitative results was done effectively with the use of participants quotes.

### Study characteristics

A total of seven studies were included in this review. The studies originated from China,^1^ the Netherlands,^35^ South Africa,^36^ Spain,^37^ Turkey,^38^ Uganda,^39^ and the United States^40^ between 1979 and 2022. Study methodologies varied and included qualitative,^35,37–40^ quantitative descriptive,^1^ and mixed-methods designs.^36^ Sample sizes of the included studies were between 7 and 1715 participants; the majority of participants were families and individual caring for ill family members,^1,35–37,39,40^ as well as college students.^38^ Each study broadly explored the role of intergenerational living on palliative and end-of-life care. Studies were performed mainly in community-based settings, including an Amish population of the United States,^40^ metropolitan areas of the Netherlands and Spain,^35,37^ and rural areas of China, South Africa and Uganda.^1,36,39^ However, one study took place in a Turkish university college setting, exploring the topic of intergenerational living in the community.^38^

### Main findings and key themes

**Figure 2.**
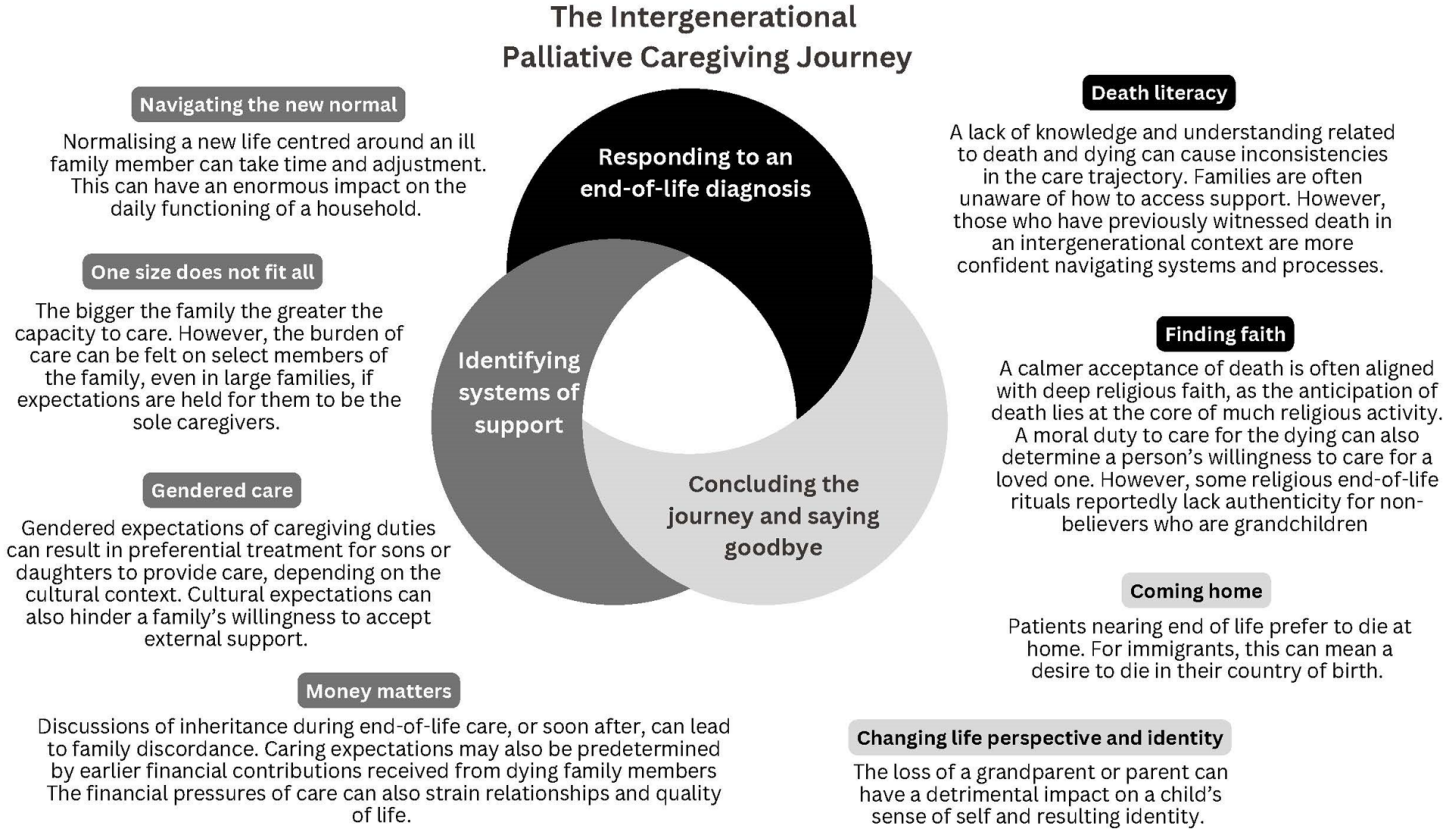
Thematic analysis.

Thematic analysis of the seven included studies identified three overarching themes that reflect shared caregiving dynamics: (1) responding to an end-of-life diagnosis; (2) identifying systems of support; and (3) concluding the journey and saying goodbye. Each main theme is supported by sub-themes, as illustrated in the thematic diagram (Figure 2). Within each theme, patterns evident across multiple cultural contexts are presented first, followed by variations shaped by cultural norms, resources, and family structures. This approach strengthens the integration across studies while maintaining sensitivity to cultural specificities. The findings are interpreted with reference to the Convoy Model of later-life family relationships, which emphasises the dynamic networks of support surrounding individuals at the end of life.

## Theme 1: Responding to an end-of-life diagnosis

This theme examines how intergenerational households mobilise emotional, practical, and spiritual resources when confronted with an end-of-life diagnosis. Across settings, families drew on varying levels of *death literacy* and religious or spiritual frameworks, shaping both preparedness for caregiving and acceptance of mortality. In Convoy Model terms, the quality of a household’s ‘core convoy’—immediate family members and close kin—was influenced by prior caregiving experience and cultural beliefs, which could either strengthen collective capacity or leave families unprepared for the demands ahead.

### Death literacy

Across multiple studies, families often faced challenges providing end-of-life care due to limited knowledge about available care services and the dying process. De Graaf and Francke’s study observed that in Turkish and Moroccan intergenerational households living in the Netherlands, the lack of awareness regarding home care services available appeared to delay the implementation of care.^35^ A family member expressed regret about the fact his mother did not die at home surrounded by her family:

The doctor might have known, that this woman didn’t have long to live anymore, and why couldn’t he inform the family [about the possibility of home care]?^35^

These households often relied on informal caregiving, until the situation became overwhelming, resulting in the need for hospital admissions and death in the undesired location.^35^ Ssengonzi’s study also reported while intergenerational caregiving in Uganda allowed families to share caregiving duties, the absence of formal education about end-of-life care left many unprepared for its emotional and physical demands.^39^

Conversely, previous caregiving experience seemed to provide families with a greater sense of confidence in managing care processes. Munthree and Maharaj discussed how caregiving responsibilities in South African households were often shared across generations, with older family members passing down caregiving knowledge to younger generations.^36^ This shared responsibility helped prepare families for future caregiving scenarios, and the intergenerational transfer of death literacy contributed to families’ abilities to navigate the complexities of care, offering a foundation for managing future end-of-life care situations.^36^ Similarly, another study found that Spanish families with previous death-related experiences were more comfortable managing palliative care, particularly when arranging home care.^37^

Bryer’s study also discussed caregiving knowledge being passed down through generations, helping family members understand and accept death.^40^ This transfer of knowledge allowed families to face death more confidently and ensured continuity in caregiving practices.^40^ One female participant stated:

We had the chance to take care of all *four* of our old parents before they died. We are both so thankful for this.

### Finding faith

A calmer acceptance of death was often aligned with deep religious faith, as the anticipation of death is central to many religious practices, in three of the included studies. Bryer highlighted that Amish families viewed mortality as a “*part of human temporality and divine eternity*,” and their religious beliefs helped them accept and cope with the loss.^40^

Karabayır and Ar-Karci found the moral duty to care for the dying, deeply rooted in religious values, was a central motivator for Turkish families.^38^ Many caregivers viewed caring for a terminally ill loved one as fulfilling a religious obligation. However, younger family members, particularly grandchildren, often found these religious practices difficult to connect with. One participant expressed ambivalence to performing a religious ritual at her grandfather’s funeral, stating:

I did not want to do it, so I could not perform it sincerely. Of course, I prayed, but I did not believe in it too much. This created confusion for me. It made me question whether I was a real Muslim.^38^

Similarly, Martin-Martin et al. also reported Spanish families saw religious faith as central to their caregiving decisions, particularly when choosing to care for loved ones at home. One wife recounted of her husband:

He was a blessed man of God. . . taking him [to a nursing home] was not an option.^37^

However, these rituals often lacked meaning for younger, non-believing family members, causing a generational divide during end-of-life care.

## Theme 2: Identifying systems of support

This theme explores how intergenerational households organise care, negotiate roles and manage resources in response to illness, including the influence of gender norms and financial obligations. The findings reveal how convoy structures are reconfigured over time, with shifts in who provides, coordinates or financially supports care. Within the Convoy Model, these adjustments represent dynamic changes to both the structure (who is involved) and the function (what kind of support is provided) of the convoy, reflecting cultural expectations, resource constraints, and interpersonal negotiations.

### Navigating the new normal

Across some of the studies, it appeared intergenerational co-residence influenced how families adapted to caregiving roles. The involvement of multiple generations in caregiving contributed to a gradual reorganisation of household duties and routines, highlighting the complex dynamics of caregiving in these households. In Ugandan families, Ssengonzi reported caregiving responsibilities often disrupted daily routines, requiring households to shift their focus towards the ill relative and adjust their daily activities and priorities to accommodate caregiving duties.^39^ This required multiple generations to contribute to the care process.^39^

Another study reported Spanish families had to gradually change their routines to revolve around the needs of an ill family member, across multiple generations, with caregiving becoming a central focus of household life.^37^ One daughter remarked:

At the beginning it was very hard, extremely hard, because you do not know what you are facing; you have fears. But we have become accustomed to each step; we have adapted.^37^

Bryer documented how Amish families faced similar challenges, with caregiving for a terminally ill loved one requiring some reorganisation of daily life.^40^ The involvement of multiple generations in caregiving altered the way families approached their everyday responsibilities, as caregiving took precedence:

Massive support, both physical and psychological, is immediately available.^40^

### One size does not fit all

According to some of the studies’ findings, in larger families the assumption that caregiving will be shared equally may not hold true in practice. de Graaff and Francke noted in Turkish and Moroccan families, despite having large extended families, the burden of care often fell on a few individuals, especially when cultural expectations played a role.^35^ Family members sometimes assumed that caregiving would be more evenly shared due to the number of people available, but this was not always the case, leading to some family members feeling overburdened:

Whenever something had to be done, it always came down to me. . . A tradition has been built up, for me doing this now. I feel like a social worker and a little nurse at the same time.^35^ (Young Moroccan mother looking after her mother-in-law and *six children)*

Despite these challenges, larger families did offer some potential benefits in caregiving. In instances where families were able to share responsibilities more evenly, the caregiving burden was reduced.^35^ This study concluded, when families communicated well and cultural expectations were more flexible, the presence of more family members allowed for more effective sharing of care tasks.^35^

In Ssengonzi’s study of Ugandan families affected by HIV/AIDS, an important dynamic emerged regarding the increasing household size when adult children returned home during their illness. Despite the apparent growth in family size, the caregiving burden disproportionately fell on elderly family members, as many of the returning adult children were accompanied by their own young children, who were too young to assist with caregiving tasks.^39^ The author reported the nuanced reality of these households was that, while they grew in numbers, the caregiving responsibilities intensified for the elderly, as they managed both the care of their dying children and the needs of their grandchildren.^39^ This created an overwhelming burden that extended beyond the physical act of caregiving to include emotional and economic challenges.^39^

### Gendered care

The synthesis of expectations in caregiving duties across different cultural contexts of intergenerational households demonstrated a distinct pattern in some of the included studies, where caregiving roles were largely influenced by gender norms. In Chinese families, Cong and Silverstein highlighted 78% of sons were expected to care for their ageing parents, especially as they approached end-of-life.^1^ The author acknowledged, this cultural expectation is deeply ingrained and aligns with the traditional value of filial piety, which emphasises the duty of sons to return home to provide care, often resulting in preferential treatment in caregiving responsibilities.^1^

In contrast, De Graaff and Francke’s study concluded caregiving responsibilities were clearly divided along gender lines.^35^ The results suggested women, especially daughters-in-law, took on the bulk of caregiving tasks such as personal hygiene, feeding and other direct care duties.^35^ In contrast, men were more involved in decision-making roles, such as communicating with healthcare professionals or making decisions about external support.^35^ The study also found that many women expressed feelings of being overwhelmed by these caregiving duties, as they were required to manage these tasks alongside other family responsibilities.^35^

Ssengonzi’s study also reinforced this gendered care pattern, identifying elderly female relatives as the primary caregivers of terminally ill family members in Uganda.^39^ The study found caregiving was viewed as inherently a woman’s responsibility, with one male participant explaining:

It is the women who give first-hand help to the sick. Women have that tender care. . . You know, these people [women] are patient in their hearts and can handle the infected very well.^39^

Similarly, Munthree and Maharaj’s research in South Africa showed a stark gender disparity in caregiving within intergenerational homes affected by HIV/AIDS.^36^ The study revealed while male family members might return home when a loved one is dying, they are less involved in direct caregiving.^36^ The study identified 42% of women, compared to only 17% of men, took on primary caregiving roles.^36^ One female lamented:

Sometimes it is my sick daughter who feels sorry for me, saying I must also take care of myself and eat.^36^

### Money matters

The financial dynamics within intergenerational households play an important role in shaping caregiving responsibilities. Cong and Silverstein highlighted the influence of financial support from parents on caregiving expectations in Chinese families.^1^ The study found that parents who provided financial support to at least one son were more likely to receive caregiving from that son later in life.^1^ Specifically, 19% of parents reported giving financial support to their sons, and only 11% provided financial support to their daughters.^1^ Sons who received financial assistance were almost twice as likely to co-reside with their parents prior to death.^1^ The authors noted financial transfers to children were a key determinant of caregiving responsibilities, thus reinforcing the idea that caregiving is often seen as a repayment for earlier financial assistance.^1^

Munthree and Maharaj investigated the financial strain in South African families caring for relatives affected by HIV/AIDS. The study found that 36% of caregivers experienced a loss of income due to their caregiving duties, while 39 cited funeral costs as a significant financial burden.^36^ These pressures disproportionately impacted women, who were more likely to assume caregiving roles due to cultural expectations, which was compounded by a lack of external support.^36^

Ssengonzi found that elderly Ugandan caregivers faced financial strain while caring for HIV-infected relatives, often exhausting their savings or selling belongings to afford care.^39^ One older father caring for his adult daughter shared:

I used to buy treatments as much as I could afford until she died. I did not receive support from elsewhere except from other community members.^39^

These financial burdens restricted access to adequate care and left caregivers in precarious situations, further compounded by the emotional and physical demands of caregiving.^39^ This dynamic reflected broader patterns of isolation and economic hardship in caregiving at the end of life.^39^

## Theme 3: Saying goodbye

This theme considers preferences for the place of death and the personal transformations that can follow caregiving experiences, particularly in younger family members. Decisions around ‘coming home’ to die often reflected the interplay between cultural values and practical feasibility, while involvement in end-of-life care could reshape identity and life priorities. From a Convoy Model perspective, these moments mark a redefinition of the convoy’s meaning and role: the convoy not only provides support to the dying person but also transmits enduring values, skills, and coping mechanisms to the next generation.

### Coming home

The preference for a home-based death was a recurrent theme across several studies, but this preference often varied depending on cultural and practical circumstances. Bryer highlighted that in Amish families, dying at home was seen as a natural part of the life cycle, where caregiving and family responsibility extended into the end of life.^40^ Families placed high value on the ability to care for their loved ones at home, which allowed them to remain in familiar surroundings during their final days.^40^ Home-based care in this context was seen as an extension of their cultural traditions of familial caregiving.^40^

In De Graaff and Francke’s study a slightly different perspective emerged. Turkish and Moroccan immigrant families in the Netherlands frequently expressed a desire to return to their country of origin for end-of-life care; a preference deeply rooted in cultural traditions, but often complicated by logistical and financial challenges, making it difficult to fulfil.^35^ As a result, many families opted for home-based care within the Netherlands, influenced by limited access to culturally competent formal care.^35^ A female participant explained, *‘when the doctor mentioned she [mother-in-law] was actually dying, he immediately added that she could not be transported anymore’*, reflecting the emotional complexity surrounding decisions of the location of death.^35^

### Changing life perspective and identity

The experience of losing a grandparent and engaging in caregiving had notable implications for the sense of identity and life perspective in younger family members. In Bryer’s study, the involvement of Amish children in the care of dying family members appeared to foster emotional maturity and an early acceptance of death.^40^ Two sisters aged 11 and 13, who were actively involved in caregiving for their dying grandfather, reflected on the experience with calmness, demonstrating the cultural norm of facing death directly in their community .^40^ Their ability to perform caregiving tasks suggested that caregiving at end of life within the family provided a framework for emotional resilience.^40^

Similarly, Karabayır and Ar-Karci’s study reported that Turkish college students experienced profound shifts in identity following the loss of a grandparent.^38^ For many, this was their first significant encounter with death, triggering existential questioning. One participant stated:

It feels like everything is meaningless. . . It triggered questions like, ‘what am I doing with my life?’ ^38^

This proximity and exposure to death and dying led to a reassessment of life choices and values, with some participants internalising traits they admired in their grandparents, such as generosity and kindness.^38^

## Discussion

This review explored how intergenerational co-residence influences end-of-life care across various cultural contexts. Using the Convoy Model of Social Relations as a framework, we interpreted the review’s findings through its three core components—structure (the size, composition and proximity of social networks), function (the types of support provided, such as emotional or instrumental), and dynamic change (how networks adapt across the life course in response to transitions like illness). Viewing the themes through these dimensions helped reveal patterns in caregiving dynamics, evolving family roles and intergenerational tensions that might otherwise remain implicit.

The Convoy Model of Social Relations, developed by Kahn and Antonucci (1980), offers a dynamic framework for understanding how individuals are surrounded by concentric layers of social relationships that provide varying levels of support across the life course. The model considers three core components: (1) the structure of social networks (e.g. size, proximity, relationship type); (2) the functions of these networks (e.g. emotional, instrumental support); and (3) the dynamic nature of relationships, which change over time depending on life circumstances and role transitions. In other words, it considers the influencing factors that govern the social relations between convoy members, such as personal characteristics (e.g. age, race, sex) and situational, life course characteristics (e.g. roles, cultural norms, and behaviour expectations of cultural groups such as family and community).^41^ This model was particularly useful in interpreting our findings by highlighting how support systems in intergenerational households shift and adapt as family members take on end-of-life caregiving responsibilities while providing the flexibility of adaptation across different cultures.^34^

The review findings suggest intergenerational living offers distinct advantages for end-of-life care, particularly in terms of emotional support, continuity of care and family cohesion. These findings align with a wider body of research, particularly in Western contexts, where the value of home-based palliative care has been increasingly recognised for improving patient and family outcomes. One study suggests home-based care in intergenerational households allows for more personalised end-of-life care, tailored to the unique needs of the family.^42^ Another study reports Western countries are re-adopting intergenerational co-residence in response to rising healthcare costs and the growing preference for dying at home.^26^

While the review and broader literature both underscore the emotional and practical benefits of intergenerational caregiving, the motivations behind these arrangements can sometimes differ. In Eastern contexts, caregiving is often a cultural obligation, driven by strong familial values and a lack of formal care services.^1,43^ In Western countries, the resurgence of intergenerational living reflects dissatisfaction with institutional care, a preference for home deaths, and the desire to maintain family continuity.^19^ The Convoy Model’s dynamic change dimension helps contextualise this trend by illustrating how caregiving networks expand or contract based on family resources and cultural expectations.^34^

While intergenerational co-residence offers important benefits, the review findings indicate it also reinforces gender norms that seem to disproportionately disadvantage women: across multiple studies, caregiving responsibilities were disproportionately borne by women. A 2021 study exploring societal perceptions of caregivers across 20 countries identified caregiving is often viewed as a woman’s responsibility, especially in more family-oriented or collectivist societies.^27^ Additionally, the study noted in some cultures where gender roles are more defined, women who provide care are often highly venerated for fulfilling these expectations.^27^ Another study exploring how gender relates to informal carers’ experiences, highlighted even in more gender-progressive societies, women were more likely to take on caregiving roles within intergenerational households, reflecting persistent gendered expectations of care.^44^ This feminisation of caregiving places significant emotional and physical strain on female caregivers, often leading to burnout and role overload.^21^ The Convoy Model’s structural component highlights how cultural norms position women in the innermost circles of caregiving networks, reinforcing expectations that they provide the most direct and sustained care.^34^ This burden, though framed as a natural or expected role, can be unsustainable without external support.^34^

The review also highlights the financial strain placed on caregivers at end of life in some intergenerational households. Through the dynamic change lens of the Convoy Model, caregiving networks may adapt to these financial pressures; however, without financial compensation or external support, caregiving arrangements may become unsustainable, particularly in cultures where this is primarily seen as a family duty.^34^ Furthermore, low and middle income countries lacking universal healthcare coverage can face even more challenges.

For instance, the theme related to gendered caregiving roles aligns with the model’s emphasis on the structural aspects of social networks, where daughters, wives, and daughters-in-law were often positioned in the innermost circles and were expected to provide the most direct care. Similarly, the intergenerational tensions described in some studies reflect the evolving nature of Convoy membership over time, especially when younger family members resist or challenge traditional caregiving expectations. The model also helped interpret the tensions between practical support and emotional availability, as these functions were not always aligned—some household members were physically present but emotionally distant, while others provided emotional support from afar. These nuances in support structure and function, as conceptualised by the Convoy Model, added interpretative depth that would likely have been missed using purely descriptive thematic synthesis.

Wider studies support the assertion that caregiving at end of life has an economic impact on the family unit, particularly in intergenerational and culturally traditional settings. In Western contexts, women are often forced to leave the workforce to provide unpaid caregiving, which deepens their economic vulnerability as they sacrifice income and career opportunities.^45^ In many societies, cultural expectations often place a double burden on women, requiring them to balance both paid employment and caregiving duties, usually without adequate formal support.^27^ Some studies argue that the integration of formal care services into family caregiving models could alleviate both the financial and emotional strain experienced by caregivers.^26,42^

Another theme identified in the review was the role of faith and reciprocity in caregiving. A recent study, exploring the concept of dignity in patients with palliative care needs, observed in Lebanese families caregiving was tied to faith, where dignity and familial reciprocity were key components of caregiving.^46^ The role of faith in caregiving at end of life highlights the cultural dimensions that influence these behaviours. For many families, caregiving is not merely an obligation but a reflection of deeply held cultural or religious values, which are critical in shaping how care is provided.^46^ The Convoy Model offers a way of understanding how these cultural values are embedded within caregiving networks, reinforcing a sense of duty and care that transcends economic or practical considerations.^34^

The review also found that intergenerational caregiving at end of life can complicate family dynamics, creating tensions around caregiving roles and responsibilities, especially in younger generations where familial responsibilities are not prioritised. This discord is framed by the Convoy Model, which suggests that caregiving networks continuously evolve as family dynamics shift.^34^ However, when communication is poor or expectations are unclear, these networks can become strained, leading to conflicts that reduce the quality of care provided and increase emotional strain on family members.^34^ One study supports this finding, noting caregiving expectations often led to intergenerational conflicts, particularly when younger generations felt unprepared or unsupported.^27^

With the application of the Convoy Model of Social Relations, we were able to move beyond descriptive categorisation and instead contextualise caregiving roles and family dynamics as fluid, socially embedded and shaped by cultural and relational histories. This theoretical lens provided a structured way to analyse the variability in caregiving support and relational burden across intergenerational households.

### Implications for future research, policy and practice

Further research is needed to explore how intergenerational co-residence at the end of life can contribute to sustainable models of care, particularly in diverse cultural and economic contexts. As highlighted by the *Lancet Commission on the Value of Death*, there is growing recognition that care models should shift from a predominantly medical focus towards community-centred approaches.^47^ Intergenerational co-residence offers a unique opportunity for family members to provide emotional, practical, and culturally attuned support. However, sustainable models ought to ensure that informal caregiving is balanced with formal support structures to prevent caregiver burnout and financial strain.

High-income countries can draw important lessons from lower- and middle-income countries, where community involvement in end-of-life care is more integrated and culturally accepted. In these contexts, death is often viewed as a collective responsibility, with families and communities actively participating in caregiving. Public health approaches in these regions emphasise social and community networks, fostering a more holistic approach to end-of-life care.^47^ By learning from these practices, Western countries can adapt community-centred models that reduce isolation and create more supportive caregiving environments for families. Additionally, frameworks like the compassionate communities movement provide a blueprint for integrating social support systems with palliative care.^48^ By embedding community networks in end-of-life care, these models ensure that caregivers are not left to manage alone, and families can access culturally appropriate and supportive services.^48^ Future research could focus on how to adapt these public health approaches to diverse cultural and economic settings, ensuring that families are supported in providing dignified and sustainable palliative care while alleviating the gendered burden of care. In intergenerational living contexts, norms around masculinity may limit male family members’ involvement in hands-on palliative caregiving, reinforcing gendered divisions of care and placing disproportionate responsibility on women, particularly older or younger female relatives.^49^ Recognising and addressing these dynamics is essential to fostering more equitable, inclusive caregiving roles and ensuring sustainable support across generations.

### Strengths and limitations

This is the first systematic review to explore how intergenerational co-residence influences palliative and end-of-life care provisions across different cultural contexts. Another key strength of this review lies in the diversity of the study team, which brought together perspectives from multiple countries and cultural contexts. This diversity enriched the analysis, particularly when examining the cultural nuances of intergenerational caregiving at end of life. By integrating insights from different cultural traditions, the study was able to offer a more comprehensive analysis of how intergenerational co-residence influences palliative and end-of-life care across varying contexts. Additionally, the review included broad publication dates and countries of origin, which allowed the exploration of the phenomena throughout different points in time and from different cultural perspectives, thereby supporting the aim of this review.

Although this study adopted a comprehensive search strategy, the challenges in describing intergenerational living arrangements for information retrieval may have led to studies being missed. Furthermore, research of importance from lower- and middle-income countries reported outside of commercial databases may not have been identified in the grey literature search based on Google searches alone. A more strategic search of the grey literature is required to determine if this is the case. Most importantly, included studies needed to explicitly describe the cohabitation of three generations. We identified many studies where this criterion was implied, perhaps in tables of participants, but not clearly stated as a factor of importance.

## Conclusion

This review highlights that intergenerational co-residence plays an important role in shaping end-of-life care across diverse cultural contexts. The themes identified suggest that cultural traditions and healthcare access intertwine to influence caregiving decisions. For many families, the decision to provide care at home appears to be as much about necessity as preference, driven by structural barriers in healthcare access.

In low- and middle-income countries, where formal healthcare options are limited, intergenerational caregiving remains essential. This offers valuable insights for high-income countries, where a shift back towards home-based care is emerging. However, the evidence suggests that while intergenerational care fosters strong familial bonds, it can impose significant burdens on caregivers, particularly women. Integrating formal healthcare services alongside family caregiving could relieve these pressures and offer a more sustainable, equitable model of care.

Future research should explore how healthcare systems can better support intergenerational caregiving, with a focus on balancing family involvement with formal support. By integrating these insights, palliative care models can evolve to meet the needs of diverse cultural and socio-economic contexts, ensuring that end-of-life care decisions are based on preference rather than constraint.

## Supplemental Material

sj-docx-1-pmj-10.1177_02692163251394880 – Supplemental material for ‘Saying goodbye’. . . A systematic integrative review of palliative caregiving in intergenerational living contexts Supplemental material, sj-docx-1-pmj-10.1177_02692163251394880 for ‘Saying goodbye’. . . A systematic integrative review of palliative caregiving in intergenerational living contexts by Madeleine L Juhrmann, Priyanka Vandersman, Raechel A Damarell, Ahmed Khamis Sharaf, Aljon San Martin, Andrew Donkor and Yakubu Salifu in Palliative Medicine
