# Supplementary material for: ‘Saying goodbye’. . . A systematic integrative review of palliative caregiving in intergenerational living contexts
Source: Palliat Med. 2025 Dec 1;40(1):31–49. doi: 10.1177/02692163251394880 (PMC12779769; doi:10.1177/02692163251394880)
Supplement: sj-docx-1-pmj-10.1177_02692163251394880 – Supplemental material for ‘Saying goodbye’. . . A systematic integrative review of palliative caregiving in intergenerational living contexts [file sj-docx-1-pmj-10.1177_02692163251394880.docx]

**Supplemental material**

**Table 4.** MEDLINE database search strategy

| # | | Searches |
| --- | --- | --- |
| 1 | | ((Intergeneration* or Multigeneration* or Inter-generation* or Multi-generation* or Cross-generation* or 3-generation* or Three-generation* or Multiple generation* or Skipped generation* or Patrilocal* or Matrilocal* or Virilocal*) adj3 (Living or House* or Housing or Residen* or Family* or Familial or Families or Support or Connection* or Caregiv* or Societ* or Solidarity)).tw,kf. |
| 2 | (Coresiden* or Co-residen* or Cohabit* or Co-habit* or "living with family").tw,kf. | |
| 3 | ((Household or family* or families or traditional) adj3 (Composition or Extended or Extension or Structure or Living arrangement*)).tw,kf. | |
| 4 | or/1-3 | |
| 5 | Extended family/ | |
| 6 | Intergenerational relations/ | |
| 7 | or/5-6 | |
| 8 | Family characteristics/ or Family structure/ or Family/ | |
| 9 | Children/ and Parents/ and Grandparents/ | |
| 10 | Adult children/ and Parents/ | |
| 11 | or/8-10 | |
| 12 | Home environment/ or Housing/ or Residence characteristics/ or Built environment/ | |
| 13 | 11 and 12 | |
| 14 | 4 or 7 or 13 | |
| 15 | (Palliat* or Terminal care or Terminally ill or "End of life" or Dying or Hospice* or Bereavement or bereaved or Advance care plan* or Advance directive*).tw,kf. | |
| 16 | ((Life limit* or Endstage* or End stage* or Advanc* or Progressive or Non-curative* or Incurabl* or Terminal or Late stage) adj1 (illness* or Disease* or Condition* or Malignan* or Heart failure or Cancer* or Organ failure or Kidney failure or Chronic kidney or Renal failure or Chronic renal or Liver failure or Chronic liver or Hepatic failure or Respiratory or Neurodegenerative or Neuro-degenerative or Chronic obstructive pulmonary disease or COPD or Dementia* or Alzheimer* or Motor neurone or MND or Multiple sclerosis or Amyotrophic lateral sclerosis or ALS or Parkinson*)).tw,kf. | |
| 17 | Palliative care/ or Terminal care/ or Hospice care/ or "Hospice and Palliative Care Nursing"/ or Palliative Medicine/ or Hospices/ or Terminally ill/ or exp advance care planning/ or bereavement/ or Death/ or Attitude to death/ | |
| 18 | or/15-17 | |
| 19 | 14 and 18 | |
| 20 | limit 19 to (arabic or english or german or hindi or tagalog) | |

*Notes: / = Medical Subject Heading (MeSH) search; exp = search on a MeSH term plus any narrower terms related to it; tw = search on title and abstract data fields; kf = search on author keywords field; adj = find terms with x number of intervening spaces between them (in either direction); * = search on all words with a common word stem.

**Table 5: Code tree**

*Palliative caregiving in intergenerational living contexts*

1. Responding to an end-of-life diagnosis

a. Death literacy

i. Lack of knowledge

ii. Intergenerational learning

b. Finding faith

i. Peaceful acceptance of death

ii. Religious obligation and intergenerational religious tension

2. Identifying systems of support

a. Navigating the new normal

i. Intergenerational co-residence

ii. Reorganisation of daily life

b. One size does not fit all

i. Cultural assumptions

ii. Disproportionate caregiving burden in larger families

c. Gendered care

i. Cultural expectations

ii. Traditional gender roles

d. Money matters

i. Financial burden

ii. Intergenerational financial support

3. Saying goodbye

a. Coming home

i. Preference for a home-based death

ii. Barriers to culturally competent care

b. Changing life perspective and identity

i. Emotional maturity

ii. Early acceptance of death
